# Membrane-Bound Configuration and Lipid Perturbing Effects of Hemagglutinin Subunit 2 N-Terminus Investigated by Computer Simulations

**DOI:** 10.3389/fmolb.2022.826366

**Published:** 2022-01-27

**Authors:** Michal Michalski, Piotr Setny

**Affiliations:** Biomolecular Modelling Group, Centre of New Technologies, University of Warsaw, Warsaw, Poland

**Keywords:** hemagglutinin, fusion peptides, MD simulations, influenza, virus

## Abstract

Hemagglutinin (HA) mediated fusion of influenza virus envelope with host lipid membrane is a critical step warrantying virus entry to the cell. Despite tremendous advances in structural biology methods, the knowledge concerning the details of HA2 subunit insertion into the target membrane and its subsequent bilayer perturbing effect is still rather limited. Herein, based on a set of molecular dynamics simulations, we investigate the structure and interaction with lipid membrane of the N-terminal HA2 region comprising a trimer of fusion peptides (HAfps) tethered by flexible linkers to a fragment of coiled-coil stem structure. We find that, prior to insertion into the membrane, HAfps within the trimers do not sample space individually but rather associate into a compact hydrophobic aggregate. Once within the membrane, they fold into tight helical hairpins, which remain at the lipid-water interface. However, they can also assume stable, membrane-spanning configurations of significantly increased membrane-perturbing potential. In this latter case, HAfps trimers centre around the well-hydrated transmembrane channel-forming distinct, symmetric assemblies, whose wedge-like shape may play a role in promoting membrane curvature. We also demonstrate that, following HAfps insertion, the coiled-coil stem spontaneously tilts to almost membrane-parallel orientation, reflecting experimentally observed configuration adopted in the course of membrane fusion by complete HA2 units at the rim of membrane contact zones.

## 1 Introduction

Influenza is a respiratory system disease that humanity has been facing since ancient times (Potter and Jennings, 2011; Lipsitch et al., 2016). A critical step in the influenza virus life cycle is its entrance to a host cell (Podbilewicz, 2014; Harrison, 2015). It involves virus internalisation through endocytosis and subsequent fusion of the viral envelope with an endosomal lipid membrane (White and Whittaker, 2016). The fusion process is mediated by viral hemagglutinin (HA) (Skehel and Wiley, 2000; Hamilton et al., 2012). It is a large, homotrimeric protein (Wilson et al., 1981; Bullough et al., 1994), whose monomers consist of two functionally distinct subunits: HA1, which is responsible for virus binding to cell receptors, and HA2, which controls the actual fusion process (Blijleven et al., 2016). HA2 subunits remain anchored in the viral membrane through their C-terminal transmembrane domains (Benton et al., 2018). Once the virus is encapsulated inside an endosome and exposed to its low internal pH, it undergoes partial refolding, resulting in the formation of an extended coiled-coil stem directed towards the endosomal membrane (Benton et al., 2020). This rigid, elongated structure is capped with flexible N-terminal HA2 regions, known as fusion peptides (HAfps), which insert into the host membrane and are directly responsible for its subsequent merger with the viral lipid envelope (Nieva and Agirre, 2003; Vaccaro et al., 2005; White et al., 2008; Apellániz et al., 2014). Notably, the role of HAfps is not merely limited to grappling the target membrane as inert anchors but most likely involves also specific interplay with bilayer structure as is suggested by their rather strict sequence conservation (Cross et al., 2009; Worch, 2014). Accordingly, there are many well-characterised point mutations that arrest or completely abrogate the fusion process in spite of preserved peptides’ ability to associate with the lipid bilayer (Steinhauer et al., 1995; Langley et al., 2009).

However, a detailed knowledge concerning structural aspects of hemagglutinin conformational rearrangements, their interactions with the membranes, or the actual mechanism of fusion and subsequent pore formation is still incomplete. The structure of the elongated coiled-coil core representing the endpoint of HA2 transformations was characterised by X-ray crystallography relatively early (Chen et al., 1999). Nonetheless, it has been long unclear whether it corresponds exclusively to a postfusion state, which occurs only after N- and C-terminal HA elements refold into a six-helix bundle, or whether it gains stability already during intermediate fusion stages, thereby serving as a scaffold for fusion peptides exposition towards the target membrane (Boonstra et al., 2018). On the one hand, this latter option was contradicted by computer simulations which indicated that the formation of an isolated coiled-coil is energetically unfavourable (Lin et al., 2018). Such a possibility would also suggest the existence of an alternative fusion pathway in which not all three peptides insert into the target membrane. However, some divert and stick to the viral membrane instead (Boonstra et al., 2018). On the other hand, rod-like HA particles of length consistent with a fully extended coiled-coil were observed by cryo-electron tomography to bridge viral and target membrane already before their close apposition (Calder and Rosenthal, 2016; Gui et al., 2016). Moreover, the presence of the complete coiled-coil was recently demonstrated in an intermediate HA structure by cryo-electron microscopy (cryo-EM) Benton et al. (2020), providing strong evidence for complete coiled-coil folding before peptides insertion into the target membrane.

In spite of those advances, the configuration of complete, trimeric HA2 N-terminus that extends past the coiled-coil remains unknown, having been not resolved either in crystal or in microscopic structures (Bullough et al., 1994; Calder and Rosenthal, 2016; Benton et al., 2020). According to experimental studies based on isolated HAfps, their N-terminal part (residues 1–11) folds into a stable *α*-helix, followed by a kink region (residues 12–14) and a second, malleable *α*-helix starting from residue 15. Early nuclear magnetic resonance (NMR) measurements, focussed on possibly shortest fusogenic peptides composed of only 20 N-terminal HA2 residues, suggested that both helices adopt a wide-open, boomerang-like geometry (Han et al., 2001). This view has been challenged by subsequent investigation of longer and more active 23 amino acid long peptides, which were shown to form a tight helical hairpin structure in micelles (Lorieau et al., 2010). It was further demonstrated that such a closed conformation actually remains in equilibrium with some fraction of more or less open boomerangs (Lorieau et al., 2012; Ghosh et al., 2015). The extent of secondary structure within HAfp is apparently not affected by further sequence elongation. As indicated by NMR, residues 24–28 form a solvent-exposed random coil, which is consistent with their belonging to a flexible tether that extends up to the coiled-coil stem (Lorieau et al., 2013). The role of this disordered region is likely to support HA2 structural integrity while HAfps remain anchored within the target membrane and the rigid stem tilts from membrane perpendicular to almost parallel orientation to fit in between two approaching bilayers (Tamm, 2003). Aside from the fact that the linker region is apparently not specifically folded such that it can withstand minor sequence elongation (up to 4 additional residues) without hampering the HA fusogenic activity (Li et al., 2008), nothing is known concerning its influence on geometries and mutual arrangement of HAfps within membrane-bound trimers.

To this end, even studies on isolated HAfps are not fully conclusive regarding their actual placement within the lipid bilayer. Once folded into a helical hairpin, two HAfp helices align in such a way that they expose exclusively hydrophobic residues on one side and more polar on the other (Lorieau et al., 2010). Such an organisation predisposes the hairpins to lie flat at the phase boundary. Indeed, most experimental evidence suggests that they remain at the lipid-water interface with the N-terminal helix partially inserted into the hydrophobic membrane core, solvent-exposed kink region, and flat laying C-terminal helical segment (Han et al., 2001; Wu et al., 2003; Lorieau et al., 2010). This view is further supported by the fact that each arm of the helical hairpin is much shorter than typical transmembrane protein helices [11 *vs*. 20 residues, respectively (Sharpe et al., 2010)]. It is not entirely clear, however, how to reconcile this picture with the evidence of membrane leakage caused by bound, but only fusion-active peptides (Wharton et al., 1988; Haque et al., 2001; Lai et al., 2005) or the observation of peptide-induced membrane area expansion that also correlates with the level of activity and not membrane binding *per se* (Longo et al., 1998; Lau et al., 2004). Moreover, NMR spectroscopy (Jia et al., 2015; Ghosh and Weliky, 2020) and several molecular dynamics (MD) simulation studies (Victor et al., 2015; Worch et al., 2017; Worch et al., 2021; Lousa et al., 2020) suggest that HAfp in helical hairpin conformation can adopt transmembrane orientation. It may be possible due to local membrane thinning induced by a favourable interaction of amphiphilic hairpin poles with an aqueous environment on both sides of the lipid bilayer. Such putative membrane-spanning configuration was shown in MD simulations to cause significant membrane perturbation and, thus, to have a much higher fusogenic potential than the surface configuration (Worch et al., 2017; Worch et al., 2021). Notably, the deeply inserted state is apparently stabilised by low pH (Lousa et al., 2020), which may provide a plausible explanation for the long-known enhancement of peptides activity by pH drop from neutral to acidic (Rafalski et al., 1991; Epand et al., 1999; Han and Tamm, 2000; Lau et al., 2004; Ge and Freed, 2009).

It remains to be clarified whether trimeric peptides arrangement enforced by their tethering to the coiled-coil stem region changes the spectrum of favourable configurations within the membrane compared to free monomers. So far, some experimental studies considered engineered trimeric HAfps assemblies devoid of full HA context (Epand et al., 1999; LeDuc et al., 2000; Leikina et al., 2001; Lau et al., 2004). The shortest constructs, composed of 20 residues long fusion peptides on top of an artificial three-helix bundle, turned out to more efficiently perturb liposomal membrane than individual peptides and, unlike them, were already active at neutral pH (Lau et al., 2004). More native-like versions, composed of full-length HAfps, complete linkers, and increasingly long fragments of HA2 coiled-coil, generally showed considerably higher activity compared to monomeric HAfps (Epand et al., 1999), and, along with the coiled-coil extension, progressively advanced from being able to cause lipid mixing in vesicles (Epand et al., 1999; LeDuc et al., 2000) and biological membranes (Leikina et al., 2001) to support full pore formation and cell fusion (Sup Kim et al., 2011). Notably, full activity of all those constructs was achieved in acidic pH, although they associate with the membrane also in neutral pH, with the coiled-coil region formed already. This further confirms that the influence of pH drop on HA activity is not limited to triggering its major conformational rearrangement. However, it stimulates the efficiency of fusion peptides by changing the way they behave within the membrane.

This work focuses on studying trimeric HAfps assembly and its interactions with an aqueous and membrane environment using molecular dynamics (MD) simulations. First, we seek to investigate the degree of conformational freedom of the peptides as they remain tethered to the coiled-coil region prior to their insertion into the membrane in order to evaluate their membrane gripping range, as well as the possibility of targeting two different bilayers, such as endosomal and viral, by individual units belonging to a single HA2. Second, we analyse HAfps arrangement once they are inserted as trimers into the membrane and the degree of lipid perturbation that they induce. Finally, we assess the possibility and consequences of transmembrane peptides insertion in trimeric configuration. In all simulations, we consider monomeric or trimeric protein systems composed of HAfp, linker region, and coiled-coil truncated at residue 49. The latter provides two full turns of coiled-coil *α*-helices, thus serving for a stable anchor point to the linkers. Because the coiled-coil in a complete HA2 is a part of an elongated rod-like structure pointing away from the target membrane, we assume that the truncation should not affect the behaviour of the HAfp-linker region. We adopt a membrane model composed of pure 1-palmitoyl-2-oleoyl-sn-glycero-3-phosphocholine (POPC) molecules. They represent a basic component used for lipid bilayer mimicry, combining mixed saturated and unsaturated chains (16:0 and 18:1, respectively) with phosphatidylcholine, which is one of the most abundant lipid heads in eukaryotic membranes (Casares et al., 2019). Although the addition of other lipid components, in particular phosphoethanolamine (PE) based and cholesterol to better reproduce the composition of endosomal membrane, may affect the details of the HA-driven bilayer fusion by changing membrane mechanistic properties (Domanska et al., 2013; Haldar et al., 2018), it is not expected to influence membrane-bound HAfp conformation (Gray et al., 1996). Given that HAfp fusogenic activity in pure POPC membranes has been experimentally confirmed (Chakraborty et al., 2017; Worch et al., 2017) and already studied in a number of atomistic simulation studies (Larsson and Kasson, 2013; Légaré and Lagüe, 2014), including our previous work (Worch et al., 2017, 2018, 2021), we find it a suitable baseline model.

## 2 Materials and Methods

In our computational study, we considered monomeric and trimeric N-terminal fragments of HA2 in fusion-active geometry, in a pure aqueous solvent and water-membrane system. The protein region comprised 23-residue long HAfp, GLFGAIAGFIEGGWQGMVDGWYG, 14-residue long linker, FRHQNSEGTGQAAD, and 12-residue long N-terminal coiled-coil fragment, LKSTQAAIDQIN. Due to the absence of HAfp and linker in available experimental HA2 structures, we modelled and assembled both regions in BIOVIA Discovery Studio 2021 (Biovia, 2021). The initial HAfp conformation was adopted as a tight helical hairpin based on NMR structure for membrane-bound peptide (PDB: 2KXA) (Lorieau et al., 2010). The linker was modelled according to the closest experimentally known fusogenic HA2 structure (PDB: 6Y5I). The trimeric coiled-coil fragment was obtained directly from fusion-active HA2 geometry (PDB: 6Y5K) (Benton et al., 2020).

Simulated systems included one or three 49-residue long protein chains, water molecules described by the TIP3P model (Jorgensen et al., 1983), and Na^+^Cl^−^ ions, which were used to construct a neutral system with a salt concentration of 150 mmol/L. In the trimeric system in water, we performed one additional simulation using a four-point OPC water model (Izadi et al., 2014) to assess whether system behaviour is not affected by force field-specific effects. In the case of peptide-membrane simulations, 234 POPC molecules were used to model lipid bilayer resulting in lateral dimensions of the simulation box ∼ 9.2 nm × 9.2 nm, and providing at least 3 nm separation between protein fragments across periodic system boundaries. Peptides and lipids were modelled with Amber99SB-ILDNP* (Lindorff-Larsen et al., 2010) and Amber Lipid14 (Dickson et al., 2014) force fields, respectively. In the trimeric system, we applied harmonic restraints between C*α* atoms for every level of the coiled-coil to maintain the structural integrity of the considered HA2 stem fragment. Harmonic force constants and reference distances were estimated by calculating fluctuations and separations of respective carbon atoms during unconstrained MD simulation (see Supplementary Table S1 for details) (Hamelberg and McCammon, 2004).

All MD simulations were carried out with the GROMACS software (Abraham et al., 2015). The systems were constructed using periodic boundary conditions. Covalent bonds involving hydrogen atoms were treated with the LINCS method (Hess et al., 1997) and simulations were propagated with a time step of 2 fs. Long-range electrostatic interactions were calculated using the particle mesh Ewald method (Essmann et al., 1995), and the cut-off for van der Waals interactions was set to 1 nm. Lipid bilayers were minimised and equilibrated under constant temperature and pressure conditions for 1 ns. Desired temperature of 310 K and pressure of 1 bar were maintained by Nose–Hoover thermostat (Evans and Holian, 1985) and semi-isotropic Parrinello–Rahman barostat (Parrinello and Rahman, 1981), respectively. Full simulation protocols included 5,000 steps of potential energy minimisation with the steepest algorithm, 500 ps thermalisation, 1 ns pressure equilibration, and 10 ns system equilibration prior to production run.

For the analysis of system behaviour in an aqueous environment, five independent MD simulations, for both monomers and trimers, were conducted for 5 *μ*s, the last 1 *μ*s of which was used for analysis. The solvent-accessible surface area, hydrogen bond, root mean square fluctuation (RMSF), and radius of gyration analyses were carried out using gmx sasa, gmx hbond, gmx rmsf, and gmx gyrate Gromacs tools, respectively. In contrast, the root mean square deviation (RMSD) matrices calculation was performed in MDAnalysis (Michaud-Agrawal et al., 2011). The secondary structure was determined by the DSSP algorithm (Kabsch and Sander, 1983). For RMSD matrices estimation, trajectories were firstly aligned to the coiled-coil region using C*α* atoms, and the RMSD was computed for both HAfp and linker domains. In the case of RMSF, trajectories were similarly aligned to the coiled-coil domain, and reference structures were calculated over a 5 ns sliding window.

For the analysis of the ability of unstructured trimeric HAfp assemblies formed in water to loosen up and release individual HAfps in a lipid environment, we conducted three simulations, 5 *μ*s each, using as starting geometries the randomly selected three out of five final structures from MD runs in the aqueous environment (see above). In each case, the CHARMM-GUI online interface (Jo et al., 2008) was used to insert the unstructured HAfp trimers into lipid bilayer slab composed of 234 POPC lipids, with the coiled-coil region pointing to the aqueous solvent. Three independent simulations were performed following our standard protocol (see above). In order to keep the HAfp assembly within the membrane core, simulations were conducted with harmonic restraint, with a force constant of 1,000 kJmol^−1^nm^−2^ and a reference distance of 1.15 nm between the coiled-coil (centre of mass of residues number 38) and the membrane midplane, defined by the centre of mass of phosphate atoms.

For the analysis of membrane-bound trimers in surface and deeply inserted configurations, the preparation of starting structures involved the adjustment of linker regions to obtain trimer geometries in which HAfps were at least 2 nm away from each other, measured as a minimal distance between respective sets of heavy atoms, to exclude their mutual interactions at the initial stages of simulations. In order to obtain the structures for surface and transmembrane HAfp locations, the axes of their N-terminal helices were either positioned perpendicular or parallel to the coiled-coil axis, respectively, and the coiled-coil axis was oriented along the *z-*axis, normal to the membrane plane. Finally, the trimers were inserted into the membrane such that the centre of mass of HAfps was at the membrane midplane. Possible collisions between protein and lipid atoms were avoided by removing overlapping POPC molecules. Sample structures after the initial 10 ns equilibration are shown in Supplementary Figure S1. The production runs for surface and transmembrane configurations in either case included a set of four simulations, 5 *μ*s each, with the last 1 *μ*s used for analysis.

The 2D maps of membrane thickness and area per lipid (APL) were calculated using the algorithm implemented in the g_lomepro program (Gapsys et al., 2013). The membrane thickness and APL values were also examined as a function of distance from the peptide. Such calculations were performed by averaging thickness and APL values in circular zones spaced by 0.05 nm and centred on HAfps centre of mass. In order to account for the trimeric system symmetry in the calculation of RMSD for C*α* atoms in HAfps trimers, we considered six possible protein chain permutations in the alignment of two structures and adopted the lowest RMSD value as the best fit (see Supplementary Table S2 for details). The penetration depth, angle between coiled-coil domain and membrane normal vector, and RMSD permutation calculations were performed in MDAnalysis. Potentials of mean force for transmembrane HAfp movement along the *z*-axis perpendicular to membrane plane were estimated based on the probability distributions of finding HAfp centre of mass, *p*(*z*), using the following relation: $G\left(z\right)=-k_{B}T\ln\left(p\left(z\right)\right)+G_{0}$ , where *k*_*B*_ *T* is the Boltzmann constant times temperature and *G*_0_ is an arbitrary constant. Uncertainty of *G*(*z*) was evaluated based on the bootstrapping analysis, during which *p*(*z*) was resampled 10^5^ times.

Lipid splays were evaluated based on the distance between carbon atoms within the lipid acyl chain and membrane surface defined by the phosphate atom of the same lipid. A splay was detected if any carbon atom was found further than 0.2 nm from the membrane centre compared to the corresponding phosphate atom. Lipids proximity to the peptide was assessed based on the closest distance of their phosphate atoms to any heavy peptide atom. We have selected lipids closer to peptides than 0.7 nm for splay analysis and, as reference calculations, we have selected lipids further than 3 nm from peptides. Water-membrane permeability, *P*, was estimated assuming inhomogeneous solubility-diffusion mechanism (Marrink and Berendsen, 1994), based on water density profile across the membrane, *ρ*(*z*), and position-dependent water diffusion coefficient in the *z*-direction, *D*(*z*):

$$\frac{1}{P}=\int_{z_{1}}^{z_{2}}\frac{\rho_{0}}{\rho \left(z\right)D\left(z\right)}dz$$ (1)

with *ρ*_0_ denoting bulk water density. Water density profiles were evaluated with gmx density tool. For the calculation of diffusion profiles, the *z*-axis was discretised into bins, *z*_*i*_, of 0.2 nm width, and if a water molecule was found within a given bin at time *t*, that is, *z*(*t*) ∈ *z*_*i*_, its displacement, *δz*_*i*_, within Δ*t* = 10 ps was used to obtain position dependent diffusion constant: $D\left(z_{i}\right)=<\delta z_{i}^{2}>/\left(2\Delta t\right)$ . Water permeability in the presence of a trimeric HAfp was calculated for the entire membrane and should be interpreted as corresponding to computational conditions of monomeric HAfp with 1:78 peptide to lipid ratio.

For bioinformatic analysis of HA2 N-terminus, HA2 amino acid sequences available in the UniProt database (Consortium, 2020) were aligned using the BLAST algorithm (Altschul et al., 1990). Sequence variability was represented by the WebLogo 3.3 server (Crooks et al., 2004). The analysis of the coiled-coil forming propensity and Shannon sequence entropy were performed in the DeepCoil (Ludwiczak et al., 2019) and Los Alamos National Laboratory HIV Sequence Database (Kuiken et al., 2003) software, respectively. Hydrophobicity (HD) analysis was performed according to Kyte and Doolittle scale using the ProtScale web service (Abraham and Leo, 1987; Gasteiger et al., 2005).

## 3 Results and Discussion

### 3.1 HA2 N-Terminus

**FIGURE 1:**
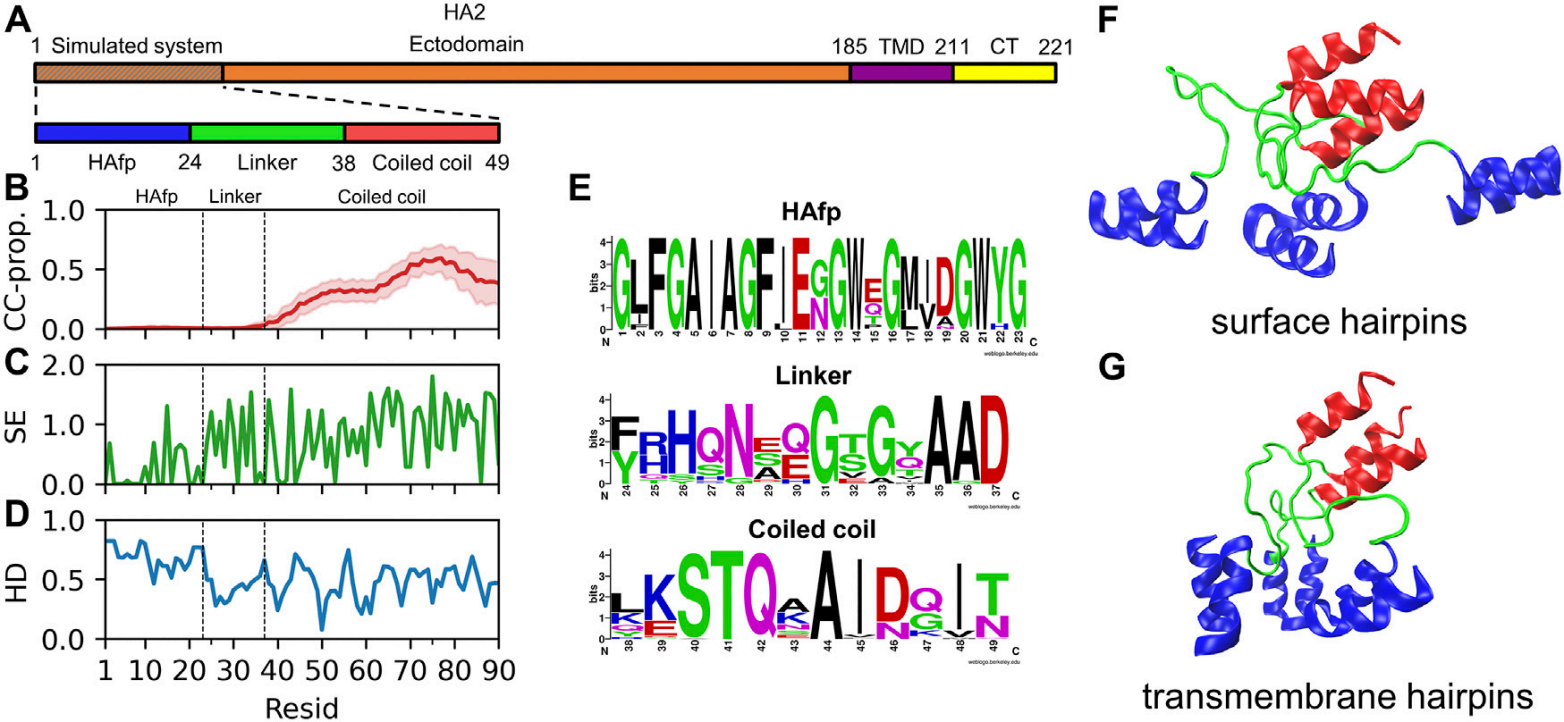
**(A)** Schematic representation of influenza virus HA2 subunit. **(B)** Mean coiled-coil forming propensity (CC prop.) of HA2 N-terminus sequence variants. Shadows correspond to one standard deviation. **(C)** Shannon entropy for HA2 N-terminus sequences. **(D)** Hydrophobicity of the considered HA2 N-terminus, normalised to span [0,1] range, along with increasing hydrophobic character. **(E)** Sequence conservation of HA2 N-terminus region. **(F,G)** Surface and transmembrane HAfp configurations, respectively, after 5 *μ*s simulation time (randomly selected one out of four simulations in either case).

In its fusion-active configuration, the trimeric HA2 subunit adopts a form of an extended 15 nm long rod, which is anchored in the viral membrane *via* C-terminal transmembrane domains (TMDs) (Benton et al., 2020). It is initially directed perpendicularly outwards from the viral surface and exposes N-terminal fusion peptides towards the endosomal membrane. The central part of the ectodomain is formed by continuous, elongated *α*-helices folded into a rigid coiled-coil. According to the X-ray (Chen et al., 1999) and cryo-EM Benton et al. (2020) structures, when looking from the C-terminus, the coiled-coil extends till residue 38 (Figure 1A). Our bioinformatic analysis of the available HA2 sequences confirms a clear lack of any coiled-coil forming propensity further towards the N-terminus (Figure 1B). Indeed, the coiled-coil appears to be definitely terminated by a distinct, so-called N-cap structure (Chen et al., 1999) formed by residues 35–37, whose high conservation within the sequence (Figures 1C,E) reveals evolutionary pressure towards preserving the integrity of this region. The region extending between residues 24 and 34, which corresponds to linkers connecting the N-cap and HAfp, does not have a particularly conserved sequence, and its structure has not been resolved in HA fusion-active state by any experimental method. Taken together with its relatively hydrophilic character (Figure 1D), it indicates that it is most likely disordered and should not have a tendency to insert into the lipid membrane. In contrast, HAfps (residues 1–23) have definitely hydrophobic character, except for the kink region (residues 11–14) and show rather high sequence conservation with notable presence of GxxG and GxxxG motifs typical for tightly interacting transmembrane helices (Russ and Engelman, 2000).

### 3.2 N-Terminus Structure and Dynamics in Water Environment

Isolated HAfps remain disordered in the aqueous environment, and they have a strong tendency to aggregate due to high overall hydrophobicity (Wharton et al., 1988; Rafalski et al., 1991). A scenario in which HA2 coiled-coil stem folds completely already before reaching the target membrane implies that HAfps at its N-terminus become exposed to water as closely bound trimers. Accordingly, it is unknown whether they retain enough conformational freedom to individually seek lipid contact or rather collapse into any particular arrangement. In order to investigate this issue, we conducted a series of 5 MD runs, each 5 *μ*s long, during which we compared the time evolution of N-terminal trimeric HA2 constructs with monomers simulated in analogous conditions in a box of water.

**FIGURE 2:**
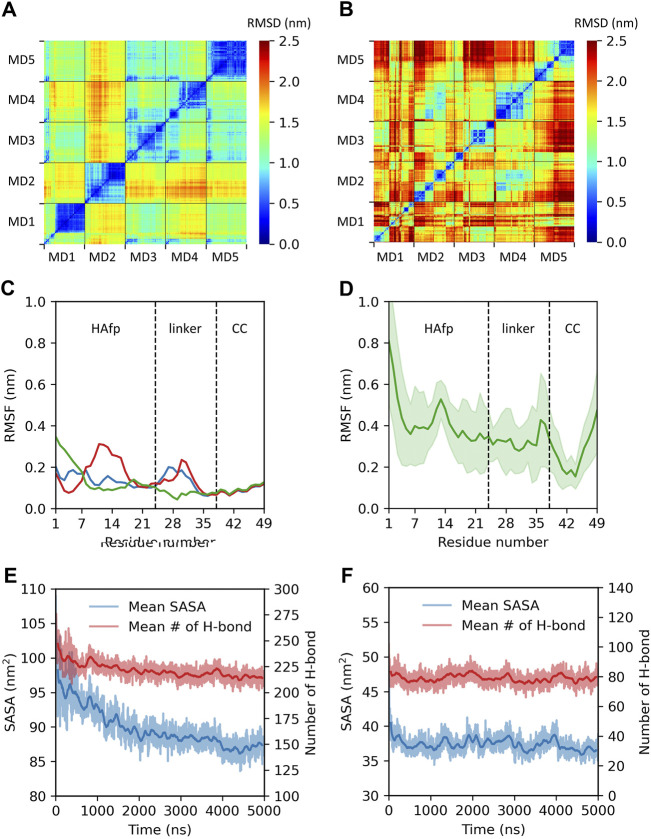
Summary of trimeric and monomeric HAfp dynamics in an aqueous environment. Two-dimensional root mean square deviation (2D cross-RMSD) of HAfps and linkers C*α* atoms following the alignment of the coiled-coil regions in **(A)** five trimeric and **(B)** five monomeric simulations. **(C,D)** Residue-based C*α* root mean square fluctuation (RMSF) profiles for HAfp trimers and monomers, respectively. CC refers to the coiled-coil. **(E,F)** Time evolution of mean solvent accessible surface area (SASA) and mean number of intermolecular protein-water hydrogen bonds during HAfp trimers and monomers simulations, respectively. Shadows correspond to one standard deviation across 5 MD runs.

All starting structures corresponded to extended configurations of HAfps attached *via* linkers to a short fragment of the coiled-coil. As illustrated by cross-RMSD matrices of HAfp-linker regions, the dynamics of trimeric (Figure 2A) and monomeric (Figure 2B) states turned out to be rather different. In the case of trimeric systems, pairwise RMSD values stabilised after initial relaxation and remained steady at the level ≲ 0.5 nm till the end of simulations, indicating the cessation of major conformational changes (Figure 2A, diagonal sub-matrices). The suppression of dynamics was apparent both within HAfps and linker regions, as is reflected by low, ∼ 0.2 nm, average amplitude of RMSF recorded within the last 1 *μ*s of simulations (Figure 2C). The final geometries in each run lacked any defined secondary structure (see Supplementary Plot S2 for details) and were mutually dissimilar, as evidenced by high (≳ 1 nm) pairwise RMSD values between individual simulations (Figure 2A, off-diagonal sub-matrices). On the contrary, the monomeric systems constantly sampled different states, and no long-term stabilisation was observed throughout the entire MD runs, with the degree of conformational variability increasing towards the N-terminus (Figure 2D). These findings are further supported by different trends in the hydration of HAfp-linker segments. In the case of trimers, a gradual solvent expulsion from the protein environment was observed, evidenced by a decrease of solvent accessible surface area and a diminishing number of protein-water hydrogen bonds (Figure 2E). Taken together, this indicates nucleation of a hydrophobic core which restricts further conformational rearrangements. The monomers, in turn, maintained a constant hydration level (Figure 2F), which suggests that a single HAfp-linker strand is too small to induce hydrophobic collapse and, thus, in the absence of defined secondary structure, remains free to constantly sample diverse geometries. Similar conclusions can be drawn from the radius of gyration and the number of intramolecular hydrogen bonds (see Supplementary Figure S3). In order to check whether the tendency of HAfps trimers to aggregate in our simulations was not overestimated due to the use of the TIP3P water model, whose bias towards the stabilisation of compact protein structures was reported in the literature (Shabane et al., 2019), we conducted an additional, analogous MD run using a more expensive, four-point OPC model (Izadi et al., 2014), which is known to increase the propensity of disordered protein states (Shabane et al., 2019). As shown in the supplementary information (Supplementary Figure S4), the tendency of HAfps trimers to aggregate remains unchanged.

We conclude from these findings that mutual interactions within three N-terminal HA2 fragments lead to their aggregation into loose, pillow-like hydrophobic assemblies on top of the coiled-coil stem (see Supplementary Figure S5). Arresting HAfps within such structures most likely limits their ability to sample space around the N-terminal HA2 cap and, subsequently, the possibility of individual, independent insertion into the target membrane. Likewise, it seems that, once the folding of the coiled stem is completed, the possibility of unproductive HA2 insertion pathways during which HAfps anchor within the viral instead of endosomal membrane would require considerable tilting of the entire ectodomain.

### 3.3 N-Terminus Structure and Dynamics in Lipid Environment

**FIGURE 3:**
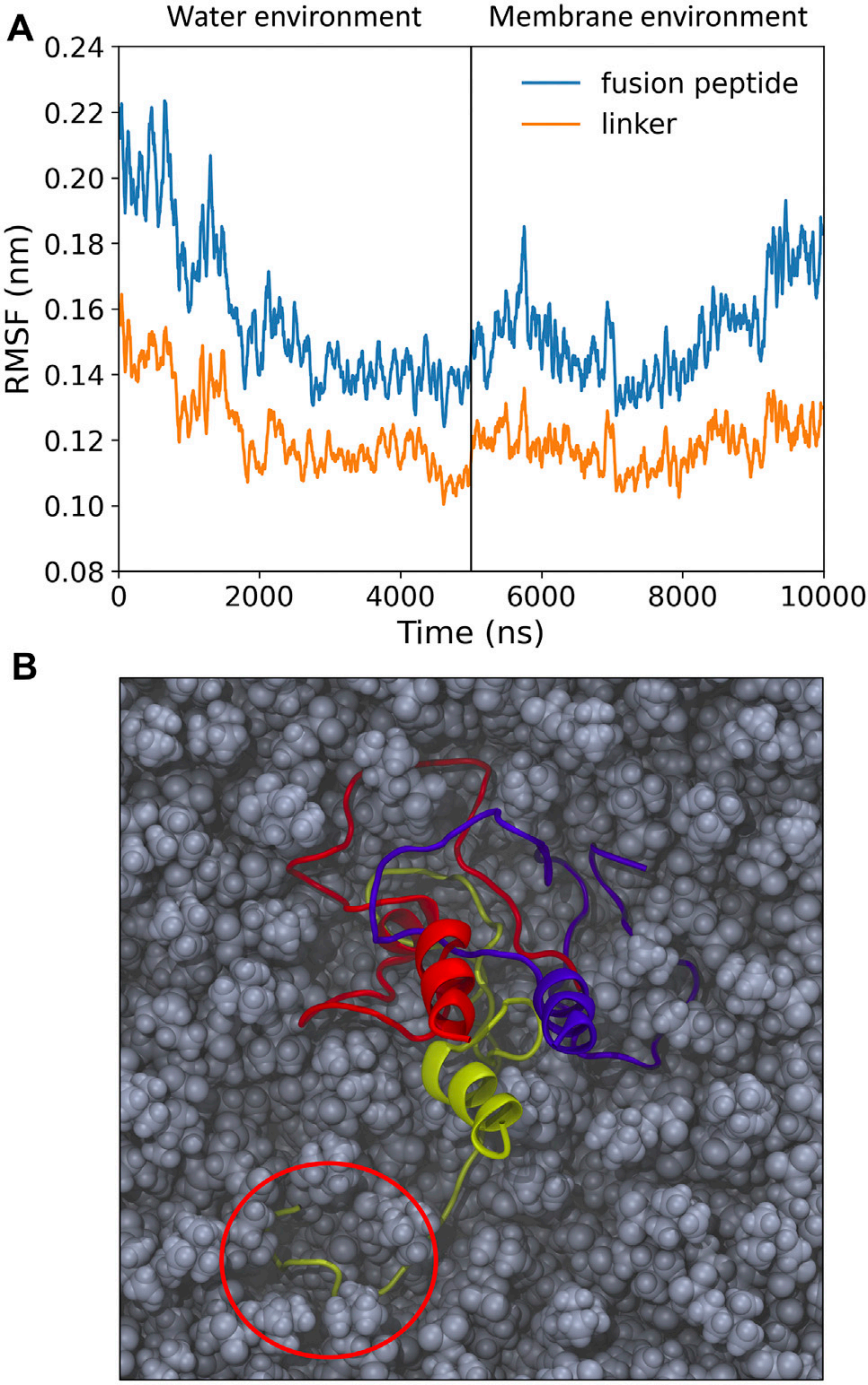
**(A)** Root mean square fluctuation (RMSF) of C*α* atoms over 5 ns windows for pillow-like hydrophobic assembly in water and following its insertion to membrane environment. **(B)** Simulation snapshot with disrupted pillow structure within lipid environment. Circle indicates a liberated HAfp with initial secondary structure.

Once inserted into the target membrane, an unstructured, pillow-like structure of HAfps trimers is unlikely to warrant HA fusogenic activity, as this is supposed to require specific helical hairpin HAfp geometry. In order to test whether a hydrophobic lipid environment is capable of inducing necessary conformational changes within the aggregates, we carried out additional simulations in which the N-terminal HA2 trimer in its final conformation from the randomly selected MD run in aqueous solvent was partially embedded within the POPC lipid bilayer. As shown in Figure 3A, an average amplitude of HAfps C*α* atoms fluctuations, which was first decreasing in water along with advancing aggregation, began to increase, indicating that the N-terminal structure indeed started to loosen up. In addition, a release of a single HAfp from the assembly and nucleation of its N-terminal *α*-helix could be already observed within 5 *μ*s simulation time (Figure 3B). Based on our previous simulations (Worch et al., 2017; Worch et al., 2021), in which a complete folding of isolated HAfps to their experimentally determined geometries at the membrane-water interface was confirmed, we believe that longer runs would indeed lead to the liberation of all three HAfps and the formation of helical hairpins.

In the following, we examine membrane-bound trimers of already folded HAfps, linked to the fragment of HA2 coiled-coil. We consider both surface-bound (Figure 1F) and deeply inserted configurations (Figure 1G), focussing on available geometries, lipid perturbation, and possible synergistic effects arising from mutual HAfps interactions.

**FIGURE 4:**
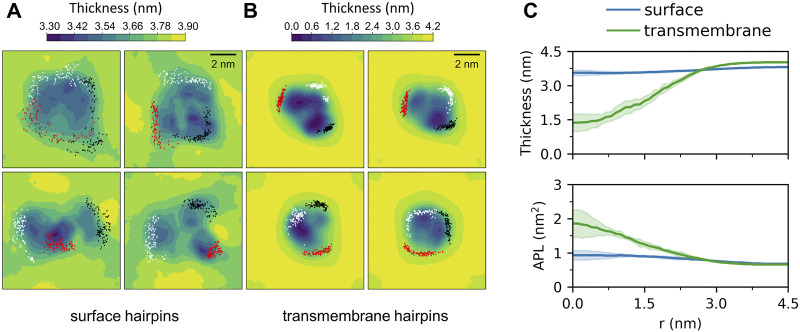
Two-dimensional maps of membrane thickness for **(A)** surface and **(B)** transmembrane HAfp configurations. Dots correspond to positions visited by N-terminal amino group of each HAfp recorded every 10 ns. **(C)** Average membrane thickness and area per lipid (APL) as functions of distance from HAfps centre of mass. Shadows correspond to one standard deviation across 4 MD runs.

**FIGURE 5:**
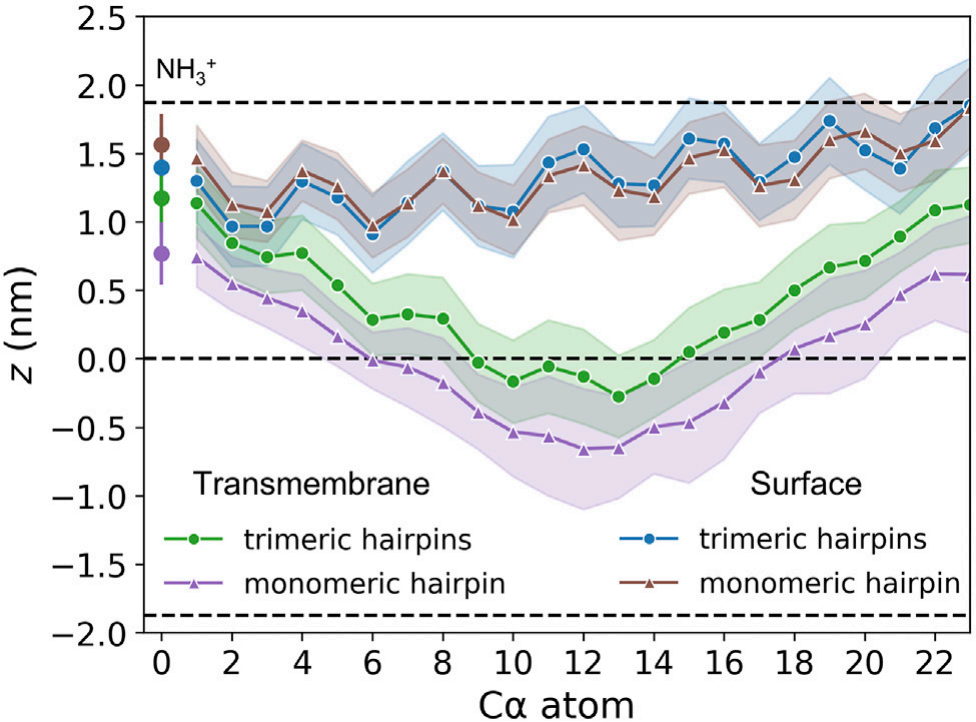
Insertion depths of N-terminal amino group nitrogen and C*α* atoms of HAfp and one standard deviation (shadow). Dashed lines represent membrane surface (the maximum of phosphate atoms density, *z* ≈ 1.9 nm) and membrane midplane (*z* = 0).

Membrane-bound HAfps display rather slow dynamics in a 5 *μ*s simulation time scale. In the case of surface configurations, each of the four independent MD runs captured different peptides arrangements and different patterns of their restricted, diffusive motion (Figure 4A). Nonetheless, as a general scheme, surface-bound HAfps revealed no tendency to associate, and each of them continuously sampled its sector of a circular area within ∼ 3 nm radius from the apex of the coiled-coil stem. An average insertion depth of their C*α* atoms was the same as observed for monomers in our previous study (Worch et al., 2021), indicating no influence of linkers or trimeric arrangement on membrane penetration (Figure 5). The perturbation of bilayer structure by surface-bound HAfps included modest thinning by up to 0.5 nm and an increase in average APL by around 50%, with both effects confined within the aforementioned circular area (Figure 4C).

**FIGURE 6:**
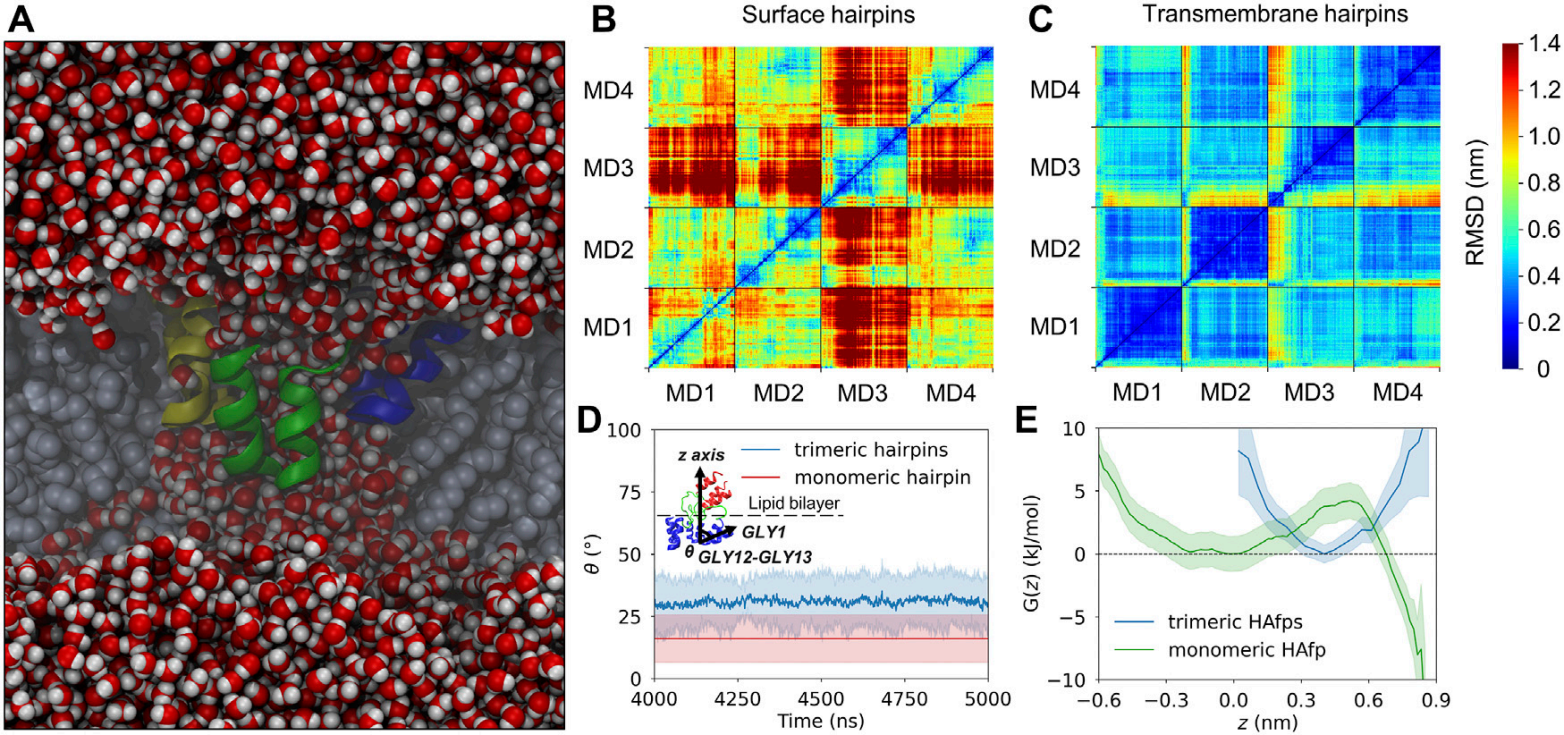
**(A)** A cross-section of the system along the *z*-axis showing a sample trimeric transmembrane HAfps (color ribbons) arrangement within POPC membrane (grey spheres) and a central channel of water molecules (red/white spheres). **(B,C)** Two-dimensional root mean square deviation (2D cross-RMSD) for C*α* atoms in HAfp trimers with optimal permutation considered. **(D)** Mean angle between HAfp N-terminal helix axis and the *z*-axis (*θ*). **(E)** Free energy profiles for HAfp movement along the *z*-axis perpendicular to membrane plane, in a free energy well corresponding to transmembrane configuration. *z* = 0 indicates bilayer midplane.

Deeply inserted HAfps exhibited even slower translational motion than surface-bound configurations (Figure 4B). Notably, however, in all 4 MD runs, each of which started from independent structures, the system of membrane-spanning peptide trimers appeared to evolve towards a distinct, common arrangement. This behaviour is evidenced by cross-RMSD matrices, which show a clear drop in mutual RMSD between individual simulations towards their ends (Figure 6C), in stark contrast to those obtained for surface-bound systems (Figure 6B). In the target configuration, all three hairpins adopt a symmetric orientation with their hydrophilic surfaces forming walls of a central, hydrated channel that extends across the entire membrane (Figure 6A). The rim of the trimeric assembly formed by HAfp kink regions is narrower than the rim formed by N-terminus such that N-terminal helices are tilted outwards by 30° ± 11° angle with respect to membrane normal (Figure 6D), and the entire trimeric structure assumes wedge-like geometry (see Supplementary Figure S6 for details). Because such asymmetric transmembrane protein profiles are known to promote bilayer curvature (McMahon and Boucrot, 2015), the observed arrangement might play a role in HA-driven target membrane protrusion towards the virus during early fusion stages as captured in cyo-electron tomograms (Calder and Rosenthal, 2016; Gui et al., 2016). As observed in the case of deeply inserted HAfp monomers (Worch et al., 2021), the trimers also induce significant membrane thinning (Figure 4B) owing to favourable interactions between amphiphilic residues at hairpin poles with aqueous solvent. The resulting membrane indentation extends up to 3 nm around the apex of the coiled-coil stem and is accompanied by an increase in APL in the affected region (Figure 4C).

The stability of the membrane-spanning configuration is hard to assess conclusively given available computational resources. Nonetheless, during 20 *μ*s of simulation time accumulated in four independent runs, none of HAfp monomers moved to the surface nor abandoned the symmetric arrangement. The free energy profile, estimated based on the probability distribution of finding HAfps centres of mass along the *z*-axis normal to the membrane plane, does not resolve the barrier between deeply inserted and surface configurations (Figure 6E, Supplementary Figure S7) in the case of trimers. Nonetheless, in comparison with the relatively shallow free energy basin for deeply inserted HAfp monomers obtained in our previous work (Worch et al., 2021), the trimeric arrangement apparently enhances the stability of membrane-spanning configuration, despite the somewhat smaller insertion depth (Figure 5).

**FIGURE 7:**
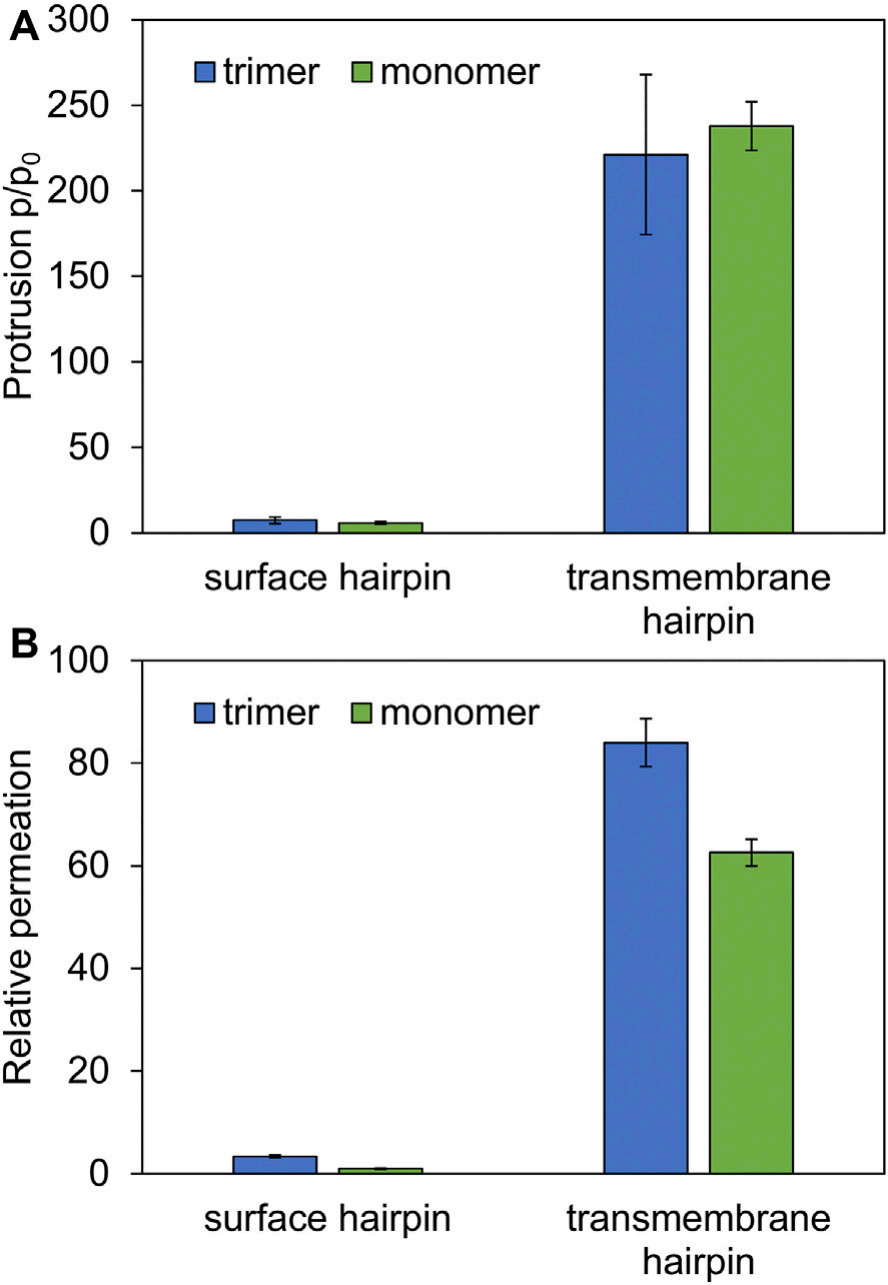
**(A)** Ratios of lipid tail protrusions for lipids within 0.7 nm distance from HAfp atoms to lipids in pure POPC membrane. **(B)** POPC membrane-water permeation for 1:78 peptide to lipid ratio in the presence of HAfp, relative to pure membrane slab composed of 78 lipids.

As might be expected, surface-bound HAfps trimers induce significantly smaller perturbation of the lipid bilayer compared to the deeply inserted configurations. One of the indicators that reflect membrane readiness to initiate lipid mixing is the intensity of lipid acyl chains protrusions (Larsson and Kasson, 2013; Tahir et al., 2016). Compared with pure POPC membrane, the probability of protrusion per lipid, per unit time within the area extending up to 0.7 nm from protein atoms was enhanced ∼ 10 times by surface-bound and ∼ 200 times by deeply bound trimers (Figure 7A). As discussed in our previous work in the context of HAfp monomers (Worch et al., 2021), the difference stems from membrane indentation caused by membrane-spanning configurations and deeper insertion of the N-terminal, charged amino group in their case (Figure 5). The enhancement of lipid tails protrusions observed here for trimers is of the same order as for HAfp monomers, suggesting that the trimerisation does not introduce any new qualitatively different effects in lipid organisation.

Another indicator of membrane perturbation is the extent of water permeability. Surface-bound HAfps trimers induced only its modest increase compared to pure POPC membrane, whereas a much more pronounced effect was observed for the deeply inserted configurations (Figure 7B). Notably, in this latter case, water permeability, normalised with respect to peptide to lipid ratio, was much higher than for membrane-spanning monomeric units, indicating a cooperative effect of trimerisation. This effect can be attributed to the fact that, in the case of monomers, a permeating chain of water molecules is supported only from one side by hydrophilic HAfp hairpin face. In contrast, in the case of trimers, the central channel is fully shielded from the membrane environment (Figure 6A). Possibly, taken together with enhanced stability of the deeply inserted configurations discussed above, this observation explains the ability of trimeric constructs to induce enhanced and less pH-dependent liposome leakage compared to monomeric HAfps, as was reported in experiments (Epand et al., 1999; Lau et al., 2004).

### 3.4 N-Cap Stability and Ectodomain Tilting

**FIGURE 8:**
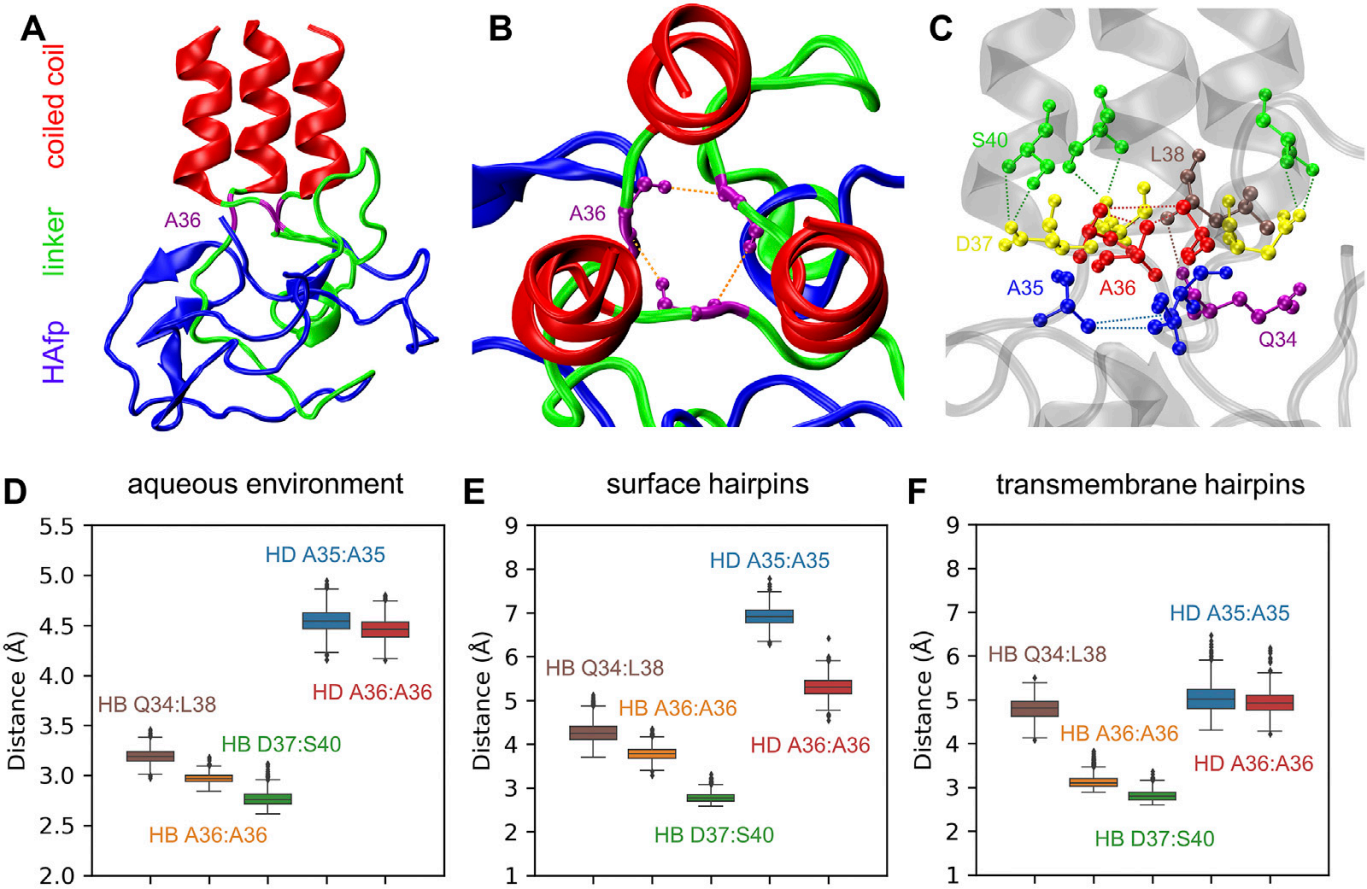
**(A)** A final structure of pillow-like HAfp assembly in an aqueous environment after 5 *μ*s simulation. **(B,C)** Main interactions stabilising the N-cap structure. **(D**–**F)** Donor-acceptor mean distances for hydrogen bonds (HBs) and methyl-methyl group mean distances for hydrophobic interactions (HD) within N-cap structures in simulations in pure water and with HAfp inserted to the lipid bilayer.

The N-terminus of the coiled-coil stem is capped by a specific structure formed by conserved residues 35–37, which apparently provides a firm base for the attachment of linkers (Chen et al., 1999) (Figures 8A–C). Major interactions stabilising this region involve a ring of hydrogen bonds between the main chain atoms of three A36 residues, accompanied by a hydrophobic cluster of their methyl groups and further supported by a second hydrophobic layer of A35 methyl groups. At the exit from this assembly towards the N-terminus, the arrangement of linkers’ main chains is further supported by the interaction of their Q34 carbonyl oxygen atoms with amide nitrogen atoms of L38 located on top of the coiled-coil helix of neighbouring monomers.

All the above interactions appear to be stable in the course of 5 *μ*s MD simulations of the trimeric HA2 N-terminus in the aqueous environment (Figure 8D). Most of them, in particular complete hydrogen-bonded and hydrophobic rings formed by A36, are also preserved once HAfps are inserted into the lipid bilayer, in both surface (Figure 8E) and transmembrane (Figure 8F) configurations. Notably, in the case of membrane-bound HA2, the coiled-coil stem tilts with respect to membrane normal (see below). It leads to the disruption of the N-cap structure below the level of the A36 ring, involving a break of at least one interaction within the hydrophobic A35 ring, as well as within the triplet of Q34:L38 hydrogen bonds (see Supplementary Figures S8–S10 for details).

At the time of initial contact between the virus and the target membrane, the distance between them is spanned by extended HA spikies (Calder and Rosenthal, 2016; Gui et al., 2016) of more than 10 nm length. The actual separation between proximal lipid leaflets at which the fusion occurs is, however, in the order of 1 nm. To enable such a close approach, HA translocates to the rim of the membranes’ contact zone, and their rigid HA2 stem structures adopt orientation almost parallel to the bilayer surface (Calder and Rosenthal, 2016; Gui et al., 2016). As the related tilting motion is executed with HAfps trimers already anchored within the target membrane, it is unclear whether the hinge within the N-terminal structure exerts any resistance and whether it has any influence on peptides insertion.

**FIGURE 9:**
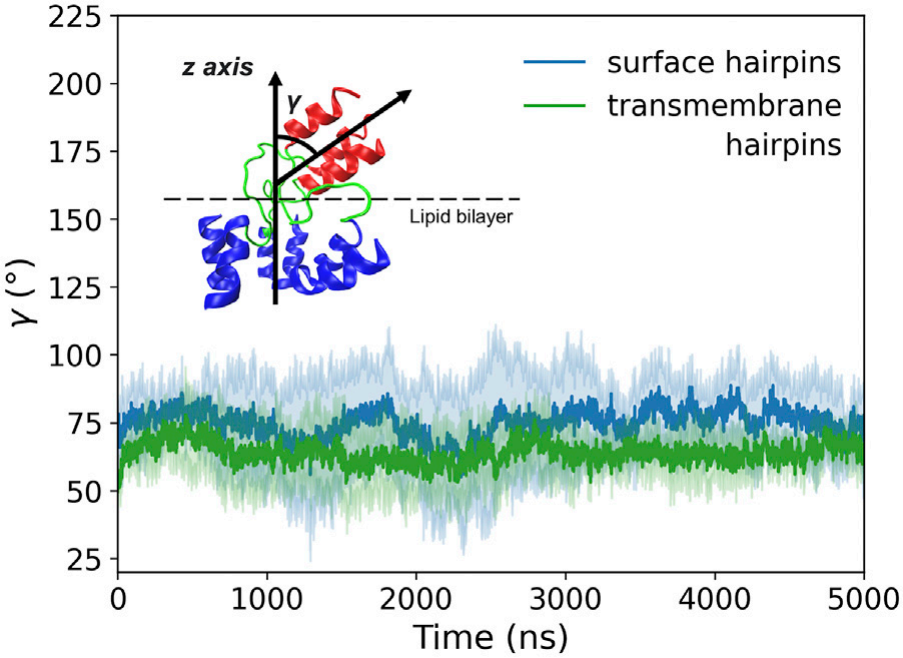
Tilt angle (*γ*) of membrane-bound HA2 N-terminus defined as an angle between the coiled-coil axis and the *z*-axis perpendicular to the membrane plane. Shadows correspond to one standard deviation.

According to our simulations, given the initially perpendicular arrangement of the coiled-coil axis with respect to the membrane plane, the tilting of membrane-bound HA2 N-terminus occurs spontaneously within the first few ns of MD run (see Supplementary Figure S11 for details), both for surface and deeply bound HAfps configurations. Subsequently, the axis of the coiled-coil region forms a stable, ∼ 70° angle with the membrane plane (Figure 9). The entire conformational change is accommodated within the unstructured linkers region. As noted above, the tilt-related symmetry breaking in this region extends up to residue A36, beyond which the N-cap and coiled-coil regain distinct, trimeric symmetry. Within our simulations time-frame of 5 *μ*s, we observed no particular influence of the tilting on HAfp structure and membrane placement neither in surface nor transmembrane configurations.

## 4 Conclusion

In this work, based on a series of MD simulations, we analysed in atomistic detail the geometry and membrane-perturbing potential of the trimeric HA2 fusogenic region. Based on experimental evidence suggesting that HA2 coiled-coil stem folds completely already before insertion of fusion peptides into the target membrane, we focussed on the arrangement of HAfps trimers tethered to stem N-terminus by linker fragments.

Our results indicate that, prior to membrane insertion, HAfps trimers have a strong tendency to collapse into compact, hydrophobic aggregates on top of the coiled-coil. The formation of such unstructured, pillow-like assemblies limits the ability of HAfp units to individually seek contact with the target membrane or to split and divert back towards the viral envelope. This observation suggests that if the coiled-coil is fully formed, fusion peptides insert jointly into the target membrane and the likelihood of alternative or unproductive fusion pathways (Boonstra et al., 2018) is limited.

Notably, once embedded within the hydrophobic lipid environment, the aggregates show a tendency to loosen their structure and release individual HAfps, enabling their folding into target helical hairpin geometries. Assuming the customary proposed peptides localisation at the membrane-water interface, we found no particular effects arising due to their tethering to the coiled-coil stem in the trimeric arrangement. Each unit sampled its own region of the membrane surface, with insertion depth and bilayer perturbing capability similar as previously determined for HAfp monomers (Worch et al., 2021). On the contrary, HAfps trimers in the transmembrane configuration were observed to form distinct, symmetric associates with a central hydrated channel. The stability of such configurations was apparently increased compared to rather metastable membrane-spanning monomers. Although, aside from enhanced membrane-water permeability, their bilayer perturbing effect was not substantially increased with respect to monomers, the stabilisation of the deeply inserted configuration alone may explain the experimentally observed greater fusogenic activity of HAfps trimers compared to monomers. Another important effect of trimerisation may be related to the wedge-like shape of the transmembrane HAfps assemblies, and the fact that their larger diameter is directed towards HA2 proximal membrane side. While not directly evidenced in our simulations due to limited system size, such an asymmetric transmembrane protein profile should promote bilayer protrusion, thus aiding in target membrane apposition to the viral envelope and increasing local curvature within the HA2 insertion zone.

Finally, our results shed light on the tilting of the coiled-coil HA2 stem with respect to the target membrane. Equally likely for surface and deeply bound HAfps, we observed no tendency of the stem to remain with its axis perpendicular to the membrane plane. Instead, we found that it spontaneously adopts a stable configuration that is almost parallel to the bilayer. Necessary symmetry breaking was confined to the linker region, with no apparent influence on HAfp dislocation nor the stability of the coiled-coil N-cap structure.

## Data Availability

The original contributions presented in the study are publicly available. These data can be found at: https://doi.org/10.6084/m9.figshare.c.5737394.v1.
