# Supplementary material for: Membrane-Bound Configuration and Lipid Perturbing Effects of Hemagglutinin Subunit 2 N-Terminus Investigated by Computer Simulations
Source: Front Mol Biosci. 2022 Jan 27;9:826366. doi: 10.3389/fmolb.2022.826366 (PMC8830744; doi:10.3389/fmolb.2022.826366)
Supplement: Supplementary file 1 [file DataSheet1.PDF]

Supplementary Material

Table S1. Harmonic force constants (k) and distances (r_0) applied to coiled coil domain and used in MD simulations of trimeric surface and transmembrane HAfps within lipid bilayer environment.

Residue number	Cα atom	Cα atom	Bond type	r_0 (nm)	k ($kJmol^{-1}nm^{-2}$)
38	522	1226	6	1.03E+00	3.30E+02
38	522	1930	6	1.03E+00	3.30E+02
38	1226	1930	6	1.03E+00	3.30E+02
39	541	1245	6	1.52E+00	2.27E+02
39	541	1949	6	1.52E+00	2.27E+02
39	1245	1949	6	1.52E+00	2.27E+02
40	563	1267	6	1.36E+00	2.39E+02
40	563	1971	6	1.36E+00	2.39E+02
40	1267	1971	6	1.36E+00	2.39E+02
41	574	1278	6	7.67E-01	6.21E+02
41	574	1982	6	7.67E-01	6.21E+02
41	1278	1982	6	7.67E-01	6.21E+02
42	588	1292	6	1.12E+00	6.08E+02
42	588	1996	6	1.12E+00	6.08E+02
42	1292	1996	6	1.12E+00	6.08E+02
43	605	1309	6	1.48E+00	5.66E+02
43	605	2013	6	1.48E+00	5.66E+02
43	1309	2013	6	1.48E+00	5.66E+02
44	615	1319	6	1.08E+00	1.08E+03
44	615	2023	6	1.08E+00	1.08E+03
44	1319	2023	6	1.08E+00	1.08E+03
45	625	1329	6	7.31E-01	1.13E+03
45	625	2033	6	7.31E-01	1.13E+03
45	1329	2033	6	7.31E-01	1.13E+03
46	644	1348	6	1.33E+00	6.55E+02
46	644	2052	6	1.33E+00	6.55E+02
46	1348	2052	6	1.33E+00	6.55E+02
47	656	1360	6	1.39E+00	7.72E+02
47	656	2064	6	1.39E+00	7.72E+02
47	1360	2064	6	1.39E+00	7.72E+02
48	673	1377	6	8.22E-01	1.06E+03
48	673	2081	6	8.22E-01	1.06E+03
48	1377	2081	6	8.22E-01	1.06E+03
49	692	1396	6	9.89E-01	1.46E+03
49	692	2100	6	9.89E-01	1.46E+03
49	1396	2100	6	9.89E-01	1.46E+03

Table S2. Possible HAfp chain permutations in RMSD calculations between reference (i) and mobile (j-o) structures. A, B, and C refer to the names of the individual HAfp trimer chain.

reference	mobile					
i	j	k	l	m	n	o
A	A	A	B	B	C	C
B	B	C	A	C	A	B
C	C	B	C	A	B	A

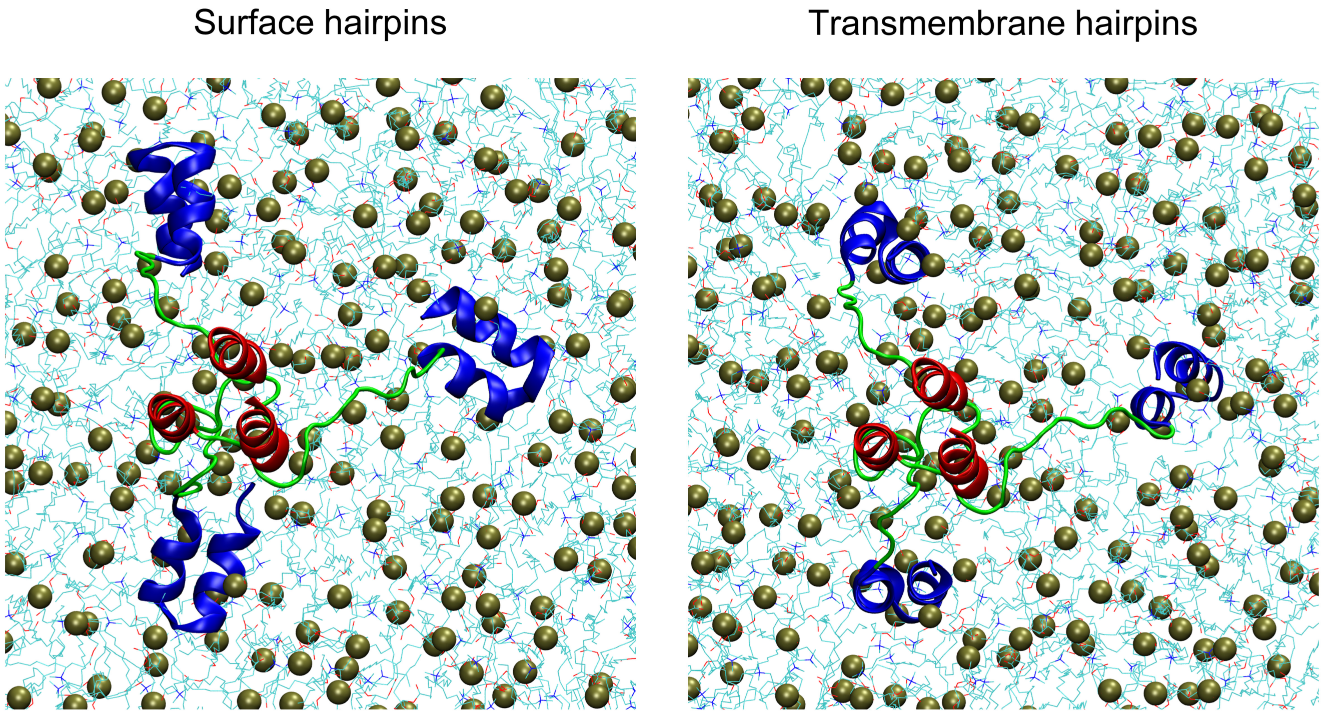

Figure S1. Starting configurations (top view on mmebrane plane) for 5 μs production runs of membrane-bound N-terminal HA2 trimers after equilibration. Proteins are in ribbon representation, lipid molecules shown as lines, phosphate atoms as spheres. Water molecules are not shown.

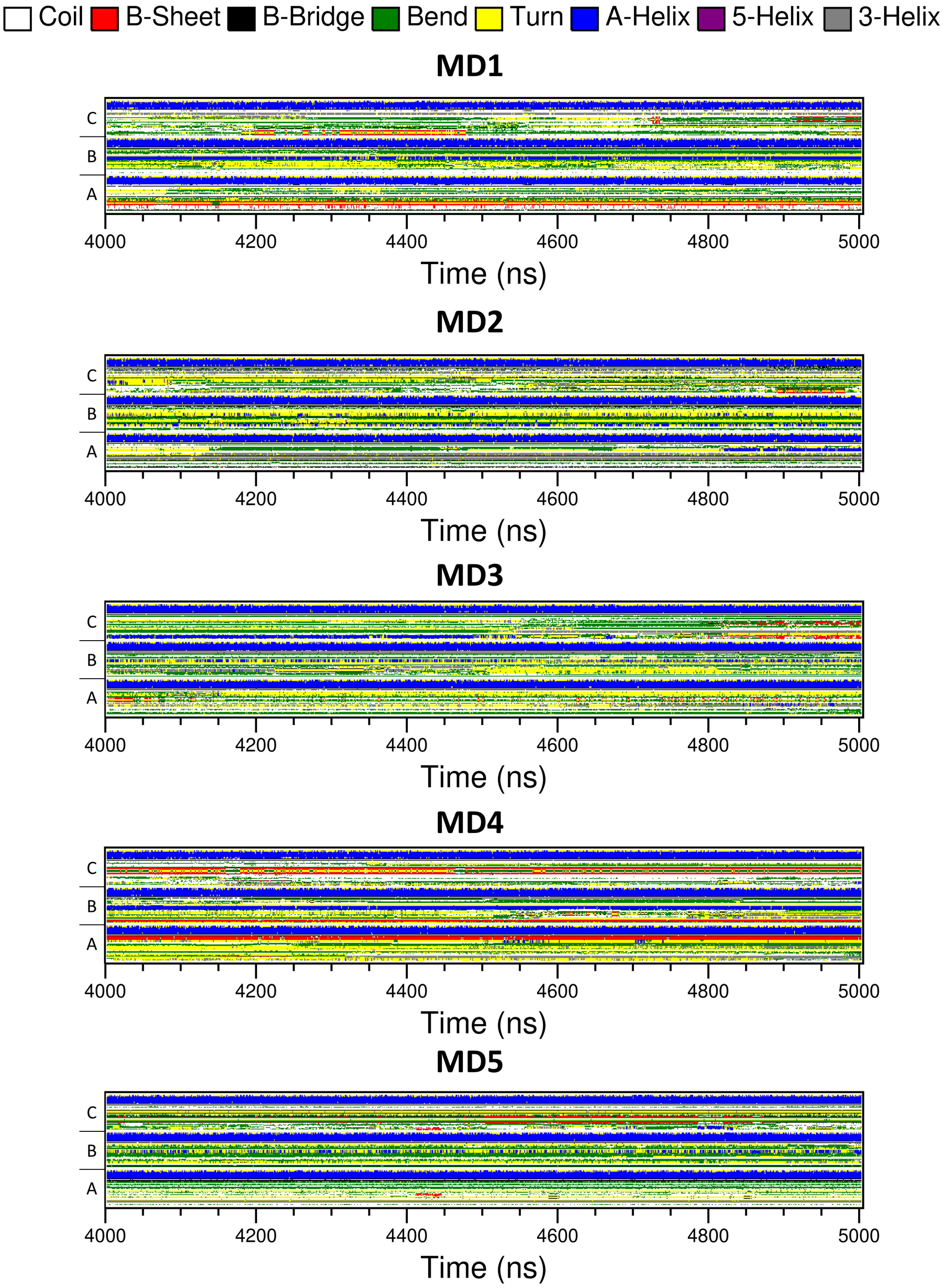

Figure S2. Secondary structure analysis based on DSSP algorithm for HAfp-linker-coiled-coil trimers in aqueous environment. A, B, and C refer to individual chains within each trimer. Aside from α-helical regions corresponding to coiled coil fragments (blue), no regular secondary structure was observed.

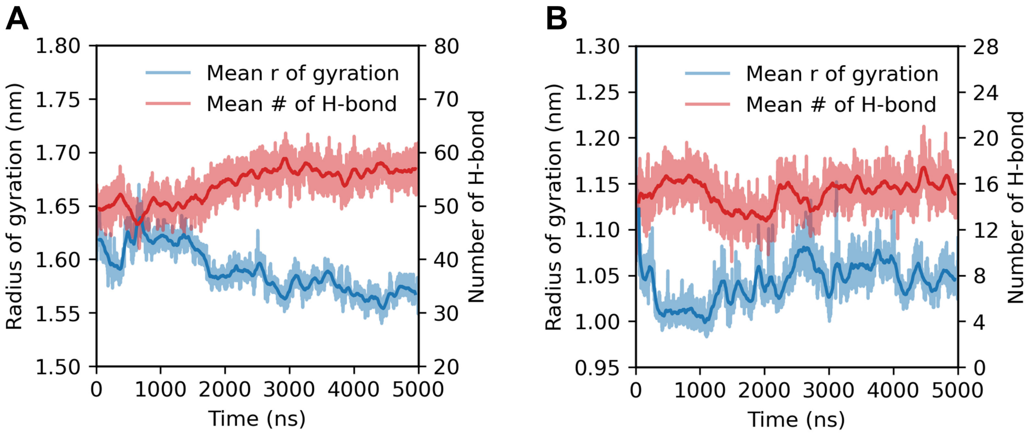

Figure S3. Radius of gyration and mean number of intramolecular hydrogen bonds during HAfp trimers (**A**) and monomers (**B**) simulations in aqueous environment. Shaded areas correspond to one standard deviation across 5 MD runs.

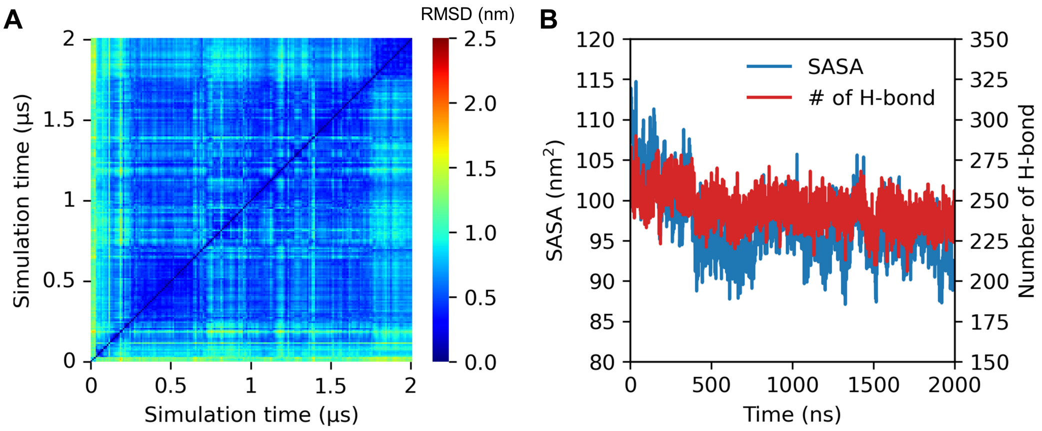

Figure S4. Results of trimeric protein structure simulation in OPC water. (**A**) root mean square deviation matrix (2D cross-RMSD) for Cα HAfp-linker domain, (**B**) solvent accessible surface area (SASA) and number of protein-water hydrogen bonds during formation of pillow-like HAfp assembly.

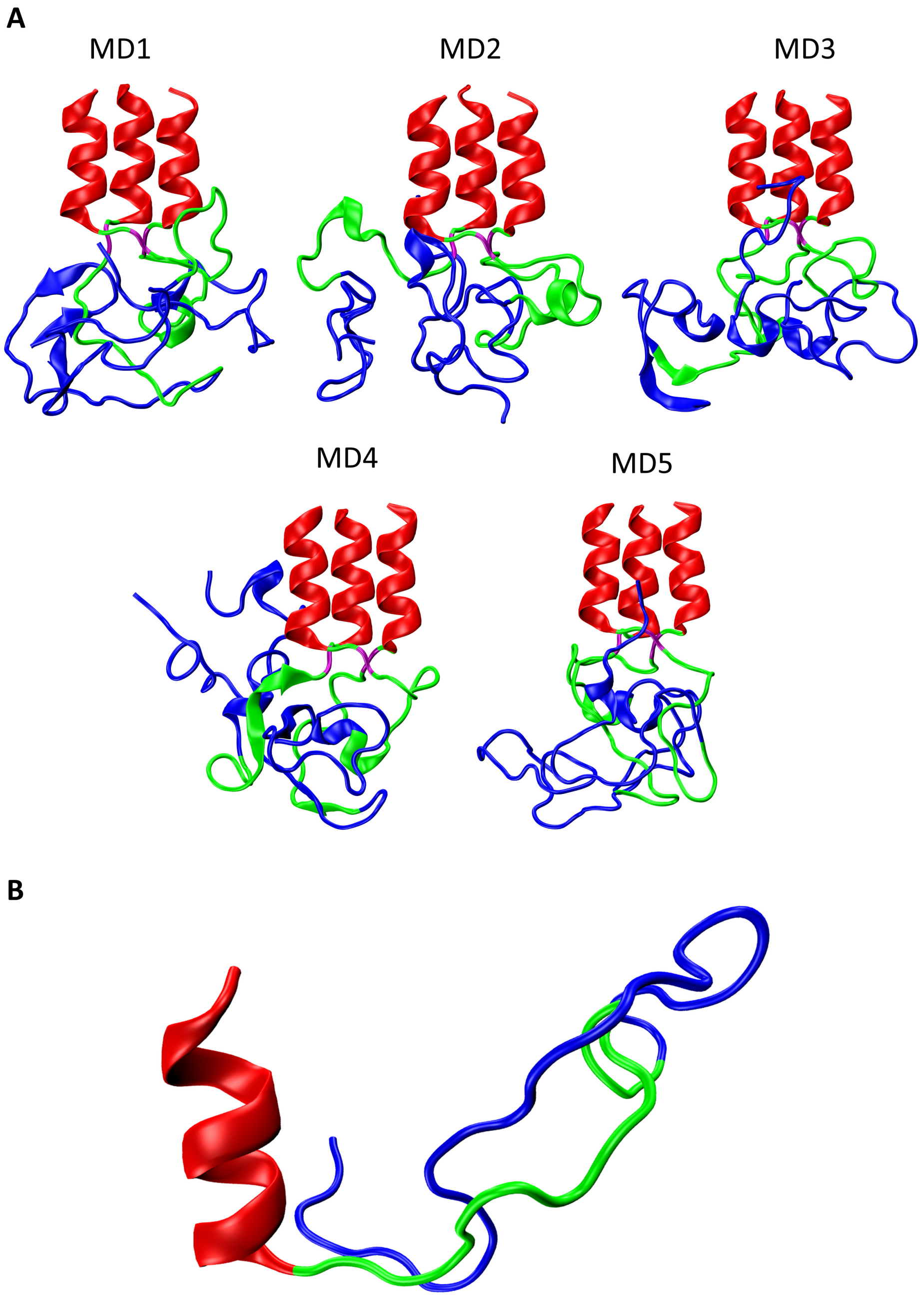

Figure S5. A set of final protein structures after 5 μs long simulations in aqueous environment (**A**) pillow-like HAfp-linker (blue and green, respectively) assemblies obtained in 5 independent simulations. (**B**) the last frame of the monomeric system from 1 out of 5 simulations (note that monomers are characterised by high conformational variability).

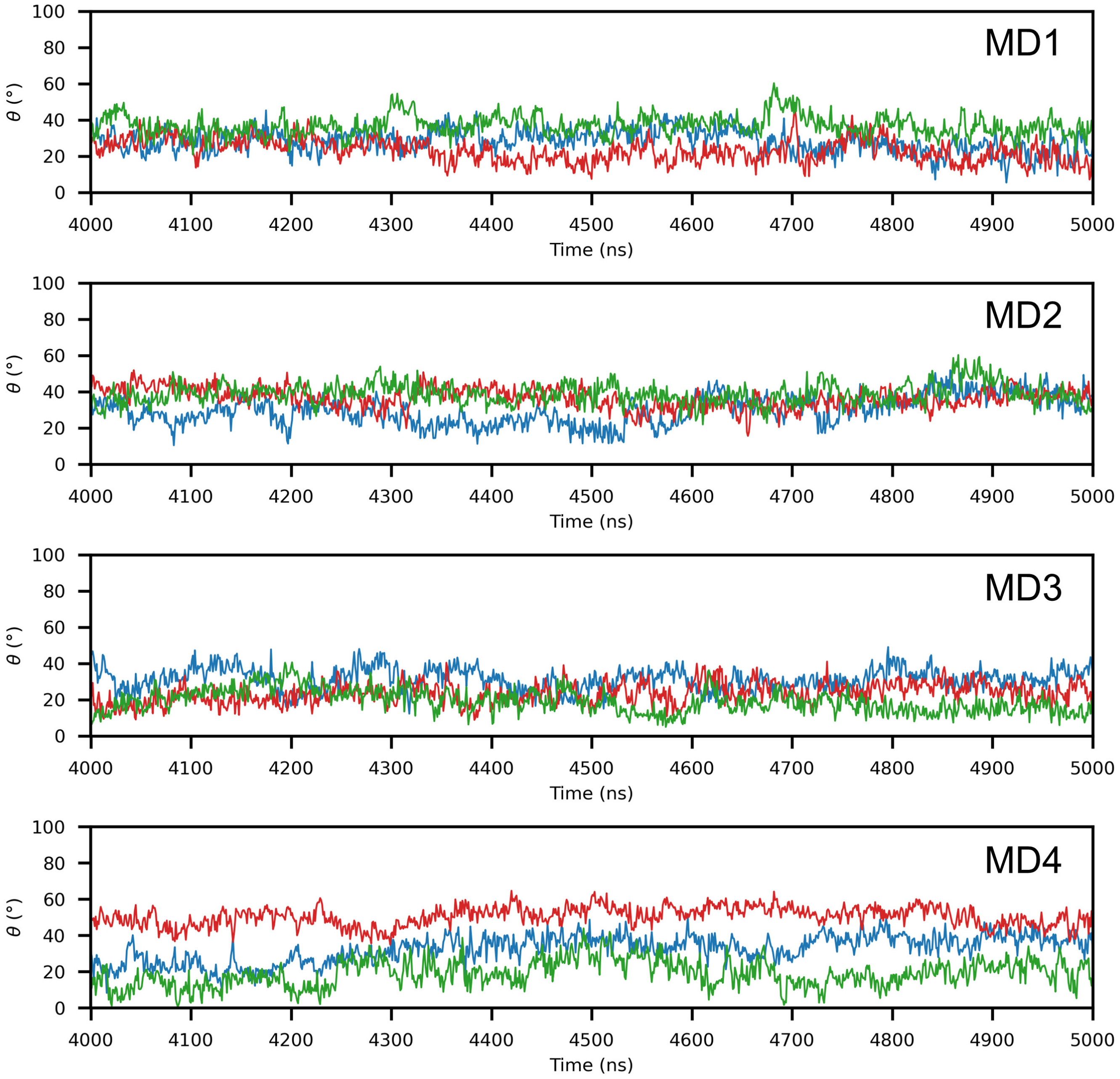

Figure S6. Simulations of HAfp trimers in transmembrane configuration: angles, θ, between HAfp N-terminal helices axes (vectors based on Cα centres of mass from residues number 1 and 12-13) and the z axis. Colors refer to individual HAfps within the trimers.

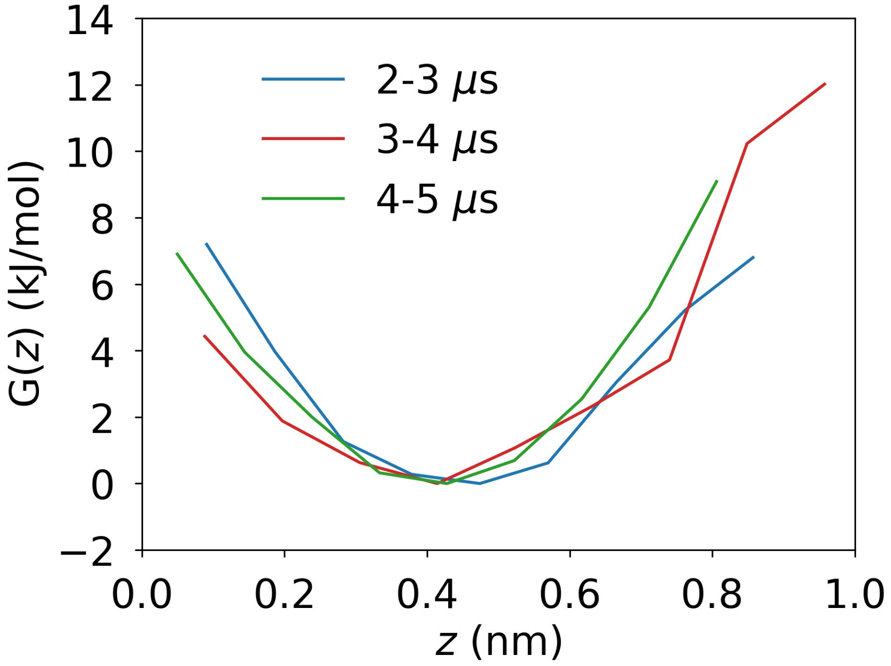

Figure S7. Potential of mean force for the movement of HAfp trimers centre of mass along the z axis in transmembrane configuration. $z = 0$ corresponds to the membrane midplane. Three consecutive simulation blocks spanning the last 3 μs are shown.

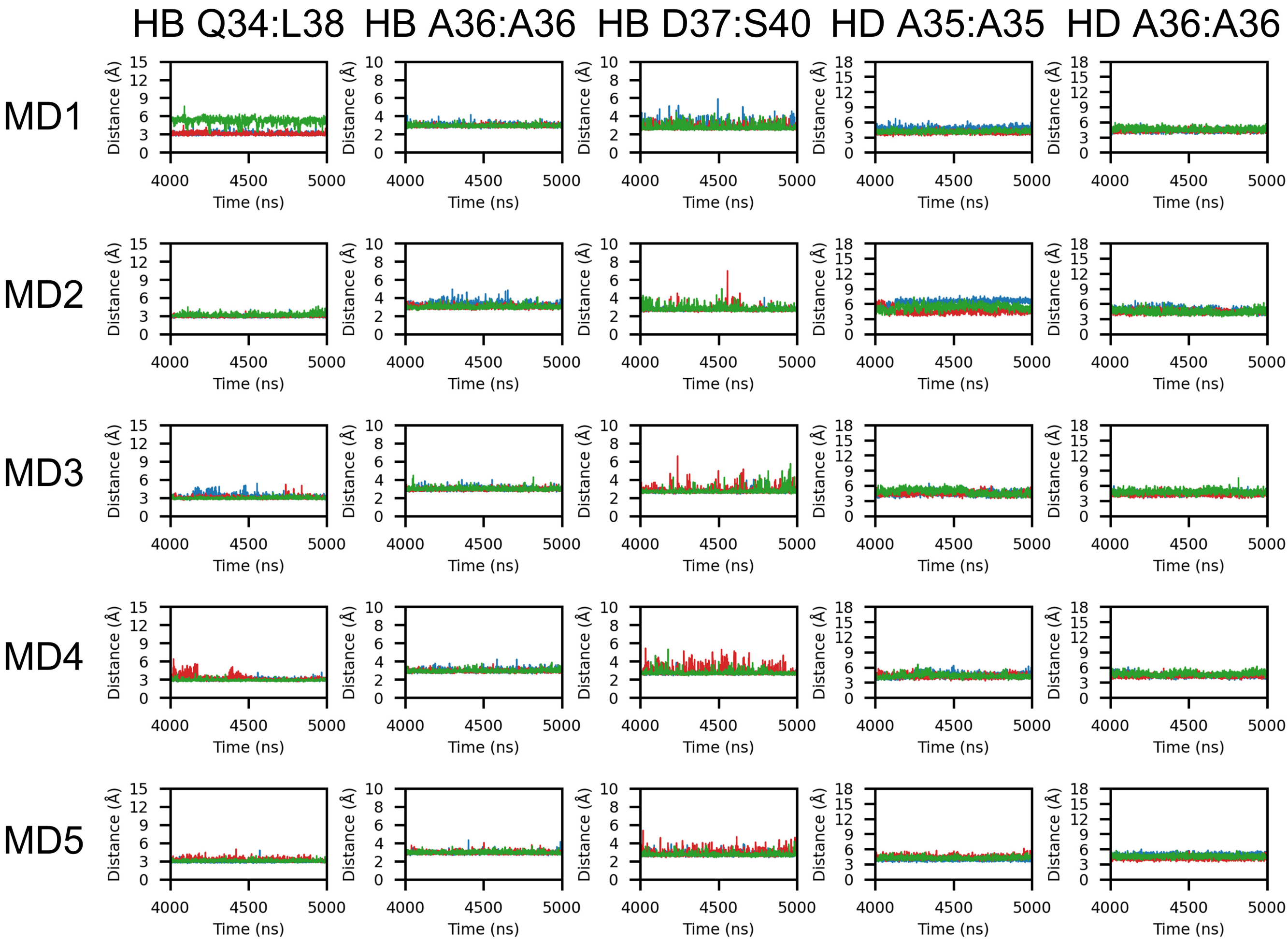

Figure S8. Donor-acceptor distances for hydrogen bonds (HBs) and methyl-methyl group distances for hydrophobic interactions (HD) within N-cap structures in simulations of HAfp trimer in pure water.

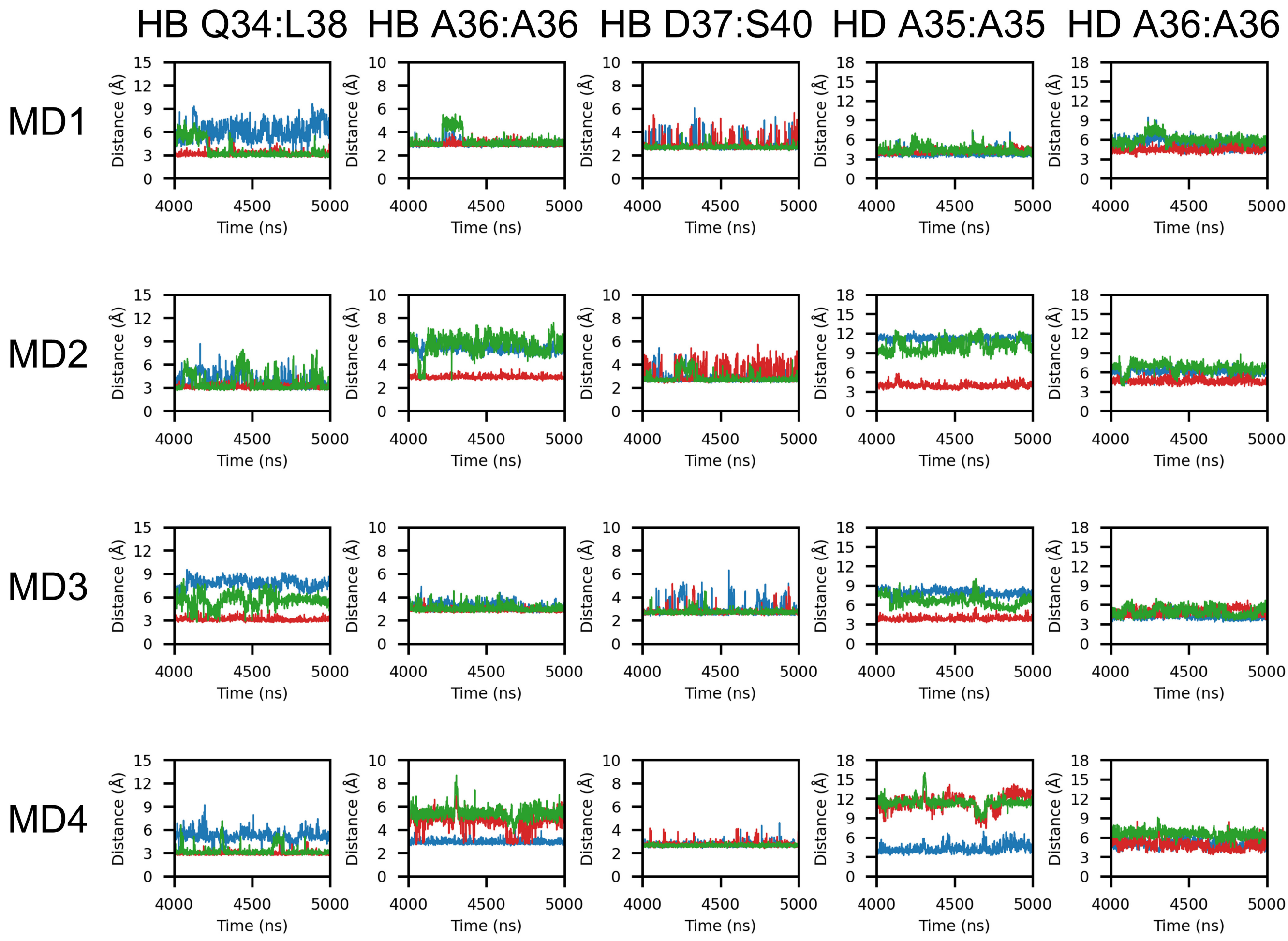

Figure S9. Donor-acceptor distances for hydrogen bonds (HBs) and methyl-methyl group distances for hydrophobic interactions (HD) within N-cap structures in simulations of surface HAfps in lipid bilayer.

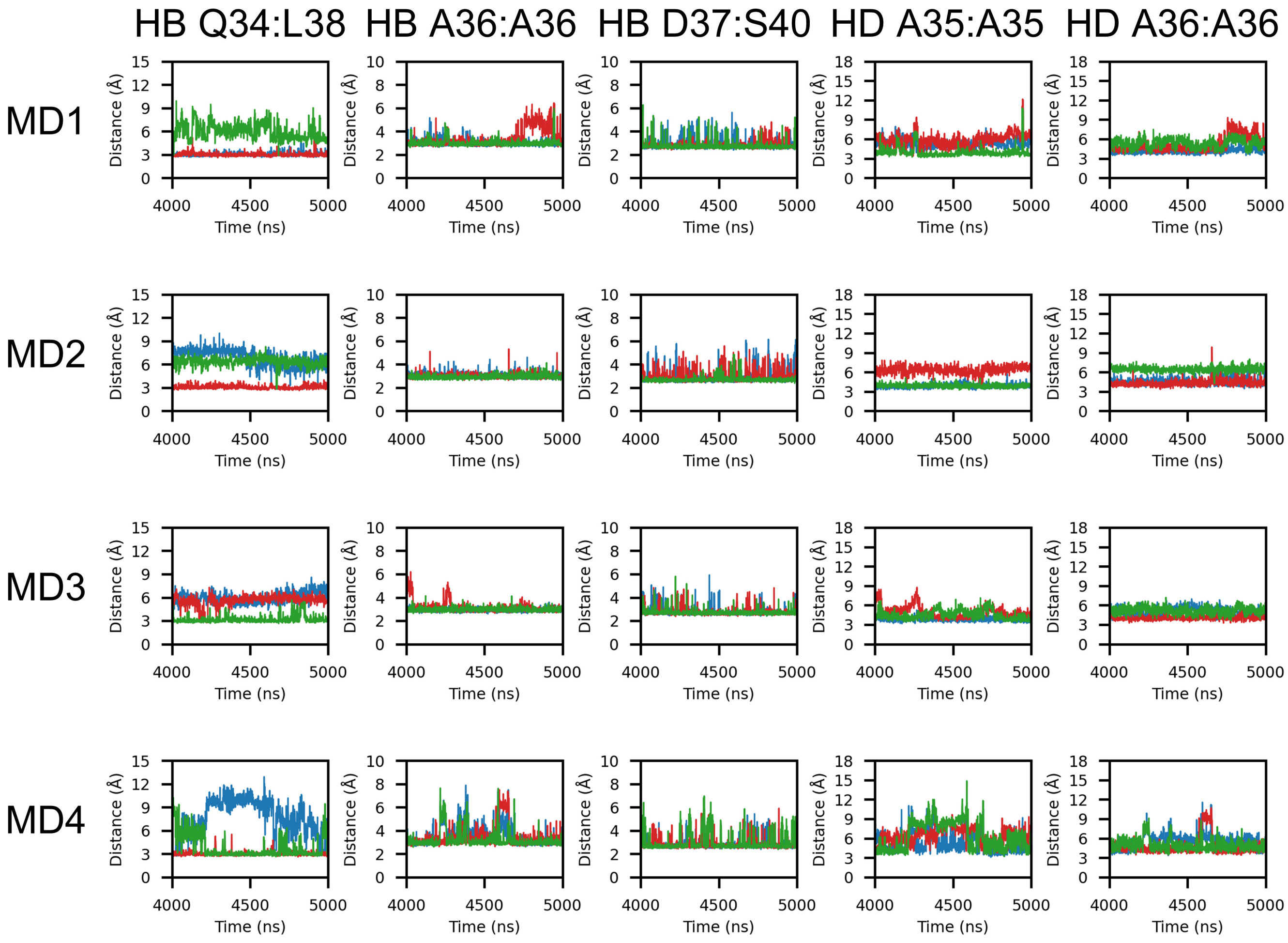

Figure S10. Donor-acceptor distances for hydrogen bonds (HBs) and methyl-methyl group distances for hydrophobic interactions (HD) within N-cap structures in simulations of transmembrane HAfps in lipid bilayer.

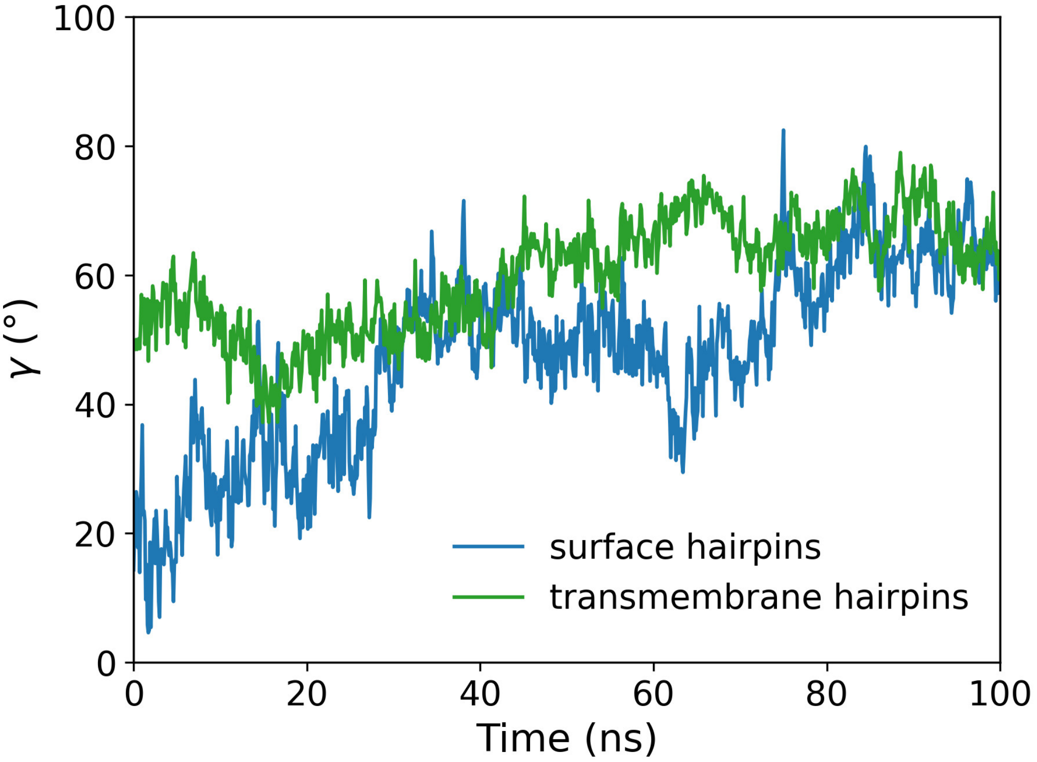

Figure S11. Tilting of membrane-bound N-terminus defined as an angle between coiled coil axis and z axis (γ), in initial 100 ns of simulations.
